# The role of FIBCD1 in response to *Aspergillus fumigatus* in lung epithelial cells

**DOI:** 10.1371/journal.pone.0282347

**Published:** 2023-03-08

**Authors:** Shreya Bhattacharya, Alec Jacob Maupin, Anders Grønnegaard Schlosser, Ernst-Martin Füchtbauer, Yamel Cardona Gloria, Alexander N. R. Weber, Uffe Holmskov, Jesper Bonnet Moeller, Steven P. Templeton

**Affiliations:** 1 Department of Microbiology and Immunology, Indiana University School of Medicine-Terre Haute, Terre Haute, Indiana, United States of America; 2 Department of Biology, Indiana State University, Terre Haute, Indiana, United States of America; 3 Department of Cancer and Inflammation Research, Department of Molecular Medicine, University of Southern Denmark, Odense, Denmark; 4 Department of Molecular Biology and Genetics, Aarhus University, Aarhus, Denmark; 5 Department of Immunology, University of Tübingen, Tübingen, Germany; 6 CMFI–Cluster of Excellence (EXC 2124) "Controlling microbes to fight infection", University of Tübingen, Tübingen, Germany; 7 iFIT–Cluster of Excellence (EXC 2180) "Image-Guided and Functionally Instructed Tumor Therapies", University of Tübingen, Tübingen, Germany; 8 German Cancer Consortium (DKTK) and German Cancer Research Center (DKFZ) Partner Site Tübingen, Tübingen, Germany; 9 Danish Institute for Advanced Study, University of Southern Denmark, Odense, Denmark; Leibniz-Institut fur Naturstoff-Forschung und Infektionsbiologie eV Hans-Knoll-Institut, GERMANY

## Abstract

Chitin, a polysaccharide, is ubiquitously found in nature and has been known to be an active immunogen in mammals, and interacts with Toll-like, mannose and glucan receptors, to induce cytokine and chemokine secretions. FIBCD1 is a tetrameric type II transmembrane endocytic vertebrate receptor that binds chitin, is found in human lung epithelium and modulates lung epithelial inflammatory responses to *A*. *fumigatus* cell wall polysaccharides. We previously reported the detrimental role of FIBCD1 in a murine model of pulmonary invasive aspergillosis. However, the effect that chitin and chitin-containing *A*. *fumigatus* conidia exerts on lung epithelium following exposure through FIBCD1 is not yet fully explored. Using both *in vitro* and *in vivo* strategies, we examined how lung and lung epithelial gene expression are modified after exposure to fungal conidia or chitin fragments in the presence or absence of FIBCD1. FIBCD1 expression was associated with a decrease in inflammatory cytokines with increasing size of chitin (dimer-oligomer). Thus, our results demonstrate that FIBCD1 expression modulates cytokine and chemokine expression in response to *A*. *fumigatus* conidia that is modified by the presence of chitin particles.

## Introduction

The lung immune response to inhalation of *A*. *fumigatus* conidia is based on innate recognition of fungal cell wall components, including β-1,3-glucan and chitin [1, 2]. In dormant conidia, these covalently linked sugars are masked by a hydrophobic rodlet layer that breaks down upon swelling and germination, thus initiating recognition via innate receptors on resident tissue macrophages, lung epithelial cells, and newly recruited inflammatory cells [3, 4]. Chitin is the second most abundant acetylated biopolymer found in nature [5–7]. Mammals encounter chitin either through ingestion, inhalation or infection with chitin-containing organisms.

A variety of pathogens and allergens express chitin and hence chitin is considered as a microbe associated molecular pattern (MAMP) in immune recognition and disease [3]. Immune recognition of chitin often coincides with recognition of β-1,3-D- glucan and mannan layers of a fungal cell wall [3, 8]. TLR2 and dectin-1 (β-glucan receptor) mediate Th17 development and neutrophil recruitment. CD206 (mannose receptor) also participates in mediating immune response to chitin.

When chitin-containing pathogens enter a host, the innate immune responses release oxidants and chitinases leading to chitin fragmentation [9–11]. The intermediate-sized oligo units fragments induce inflammation through NF-KB signalling [12]. More fragmentation continues and smaller particles of chitin are generated that induce anti-inflammatory effects through IL-10 production to control local inflammation [5]. Hence, different sizes of chitin initiate different immune responses. The difference in inflammatory responses stimulated by different sized fragments is due to combined recognition of TLR2, dectin-1, and mannose receptors [12].

Chitin also activates innate type 2 inflammatory immune responses, mainly through chitin-induced alternatively activated macrophages. Eosinophils are also recruited, leading to Th2-skewed inflammation [5, 13]. Previous results from our lab suggested that increased fungal chitin promotes eosinophil recruitment and pathology in invasive aspergillosis [14]. However, chitin can also regulate adaptive type 2 immune responses in an *in vivo* allergy model where chitin blocks allergen-induced IgE via IFNy, induced by NK cells, thereby making it a strong Th1 adjuvant [14–17]. Although these studies have described chitin-induced immune responses, the molecular mechanism of chitin recognition in lung epithelium remains elusive.

FIBCD1, a Fibrinogen-related C domain containing pattern recognition molecule, has been identified as a candidate receptor specific for chitin, although its role in fungal allergy and immunity has not been fully characterized. FIBCD1 is a membrane-bound protein that is expressed by epithelial cells lining lungs, gut, and other organs. FIBCD1 is known to bind acetylated structures, like chitin, in a calcium-dependent manner. FIBCD1 does not bind to any structures closely resembling chitin probably due to the spatial organization of the conserved FIBCD1-FReD (Fibrinogen-Related Domain) and enables endocytosis of bound ligands [18–21].

FIBCD1 binds chitin-rich regions of the opportunistic pathogen *Aspergillus fumigatus* and modulates immune responses to cell wall components of the fungus *in vitro* in a human epithelial cell line [18]. FIBCD1 has been shown to directly bind *Candida tropicalis* through exposed chitin and regulate intestinal fungal composition and inflammation *in vivo* [19]. But so far, the role of FIBCD1 in lung immune responses to chitin remains poorly understood. This study aims to examine how FIBCD1 modulates responses to chitin and chitin-containing *A*. *fumigatus* in FIBCD1-expressing lung epithelial cells and FIBCD1-deficient mice.

## Materials and methods

### Growth and handling of fungi

The clinical isolate of *A*. *fumigatus* Af293 was purchased from the Fungal Genetics Stock Center, and CEA10 was provided by Dr. Robert Cramer [Geisel School of Medicine at Dartmouth]. Isolate Af293 was cultured on malt extract agar (MEA) and CEA10 was cultured on growth minimal media (GMM) plates. The environmental isolate of *A*. *fumigatus* NRRL5517 (Af5517), obtained from the United States Agriculture Research Service, was cultured on MEA. Conidia were isolated from culture plates kept at 24°C or 37°C for 14 days by applying and gently shaking 1g of glass beads (0.5 mm, BioSpec Products), then placed in suspension by pouring the beads into a tube with sterile phosphate buffered saline (PBS). The beads were then vortexed and the supernatant containing the conidia was removed, diluted, and counted with a hemocytometer for further experimental procedures. In some experiments, harvested conidia were swollen in RPMI (Roswell Park Memorial Institute) media for 4 hours at 37°C and in some groups, were allowed to germinate in RPMI 1640 (Life Technologies) for 5 or 24 h and then fixed for 4 to 8 h in BD Cytofix buffer (BD Biosciences). Fixed, swollen conidia were washed with 0.1M ammonium chloride and PBS and resuspended in PBS for mouse aspiration or *in vitro* stimulation or surface staining and flow cytometric analysis

### Handling of mice

FIBCD1-/- mice were generated against Wild-type C57BL6 mice by Holmskov Lab, by targeting exon 2 of FIBCD1 [22]. The heterozygous mice were inter-crossed to produce homozygous wild type and Fibcd1-/- offspring. Fibcd1-/- mice were bred at the IUSM-Terre Haute animal facility with offspring used in subsequent experiments at 7–10 weeks of age. Wild-type C57BL6 mice were obtained from Jackson Laboratory and were allowed to rest 2–4 weeks prior to experiments.

### Fungal and chitin aspiration, infection, and lung harvest

Custom-sized purified chitin particles were obtained from Elicityl, ranging from chitinbiose to chitinhexaose [10 mg]. Chitin oligomer (C10-15) was generated as described previously [12]. Purified chitin particle suspensions were delivered by involuntary aspiration to isoflurane-anesthetized mice. To assess size dependent innate immune responses to purified chitin particles, mice were sacrificed 6 hours post challenge. Suspensions of 5 x 10^7^ conidia were delivered by a single involuntary aspiration. Mice were sacrificed 24 hours post-challenge with sodium pentobarbitol as described previously [17], and lungs were perfused with 10ml phosphate buffered saline (PBS) for quantitative RT-PCR (qRT-PCR) assay. Bronchoalveolar lavage was performed to collect samples to assay immune cell composition by flow cytometry. All animal procedures were approved by the Animal Care and Use Committee of Indiana State University, the host campus of IUSM-Terre Haute. All animal handling and experimental procedures were performed in accordance with the recommendations found in the Guide for the Care and Use of Laboratory Animals of the National Institutes of Health. This study was carried out in accordance with the recommendations of the PHS Policy on Humane Care and Use of Laboratory Animals. The protocol was approved by the Indiana State University Animal Care and Use Committee, the host campus of IUSM-Terre Haute. IUSM- Biosafety committee approved of the use of human cell line (approval number: SM-847-06). Indiana State University IACUC committee approved of the use of murine studies (approval number: 1507790).

### Lung epithelial cell culture

Human lung epithelial cells, A549, with over-expressed FIBCD1 were used for this study and A549-sham transfected cell line were used as controls. The FIBCD1-transfected A549 cells that we used in our study were previously characterized and described in a 2018 paper in Frontiers in Immunology [18]. A549 cells constitutively express low or non-detectable levels of FIBCD1 protein. Hence FIBCD1 expression was increased by transfecting A549 cells with the full-length human fibcd1 gene. Sham-transfected A549 cells were used as control. In A549 FIBCD1-transfected cells, FIBCD1 protein expression was confirmed by Western blot analysis and surface expression by flow cytometry by Holmskov Lab. Western blot of cell lysate from sham- and FIBCD1-transfected A549 cells under reducing and non-reducing conditions showed the presence of four known oligomeric forms of FIBCD1 in the FIBCD1-transfected cells when probed with the anti-FIBCD1 mAb clone HG-HYB 12–2. Flow cytometry revealed surface expression of FIBCD1 on FIBCD1-transfected A549 cells and none on sham-transfected cells when using anti-FIBCD1 antibody HG-HYB 12–5 for detection. These A549 cells were maintained in serial passages at 37°C, 5% CO2 humidity in RPMI media supplemented with 10% FBS, 2 mM L-glutamine, 250 μg/mL hygromycin B, 50 U/mL penicillin, and 50 μg/mL streptomycin. When confluent, cells were subcultured by washing twice in sterile DPBS, detaching with 1 mL 0.5% trypsin/EDTA/sterile DPBS, and diluted using fresh complete media. The same A549-FIBCD1 and Sham- transfected cells were used for our current study and were maintained using the same protocol. For experiments, 1 × 10^6^ cells/ml were seeded overnight in no serum growth media to arrest growth. Next day, media was replaced with regular serum media alone or co-incubated with purified chitin particles of different sizes or 5 × 10^6^ Af 293 or Af5517 or CEA10 conidia for 6 hours. Post incubation, cells were lysed in Trizol. Total RNA was isolated with Qiagen RNAeasy columns and used for cDNA synthesis. The cDNA was analyzed by qRT-PCR for determination of gene expression levels.

### Flow cytometric analysis of bronchoalveolar lavage fluid

BALF cell composition was determined by flow cytometric analysis of recovered lavage cells in suspension and stained with surface markers. In brief, BALF was centrifuged for 2 min at 2000 rpm, the supernatant removed, and the cell pellet resuspended and washed in 1 ml of FACS buffer (Phosphate Buffered Saline, 5% fetal bovine serum, 0.05% sodium azide). The washed pellet was resuspended and stained in a solution containing FACS buffer with 10% rat serum, Fc-receptor blocking antibody (clone 24G2) and the following antibodies [BD BioScience]: rat-anti-mouse Ly-6G-PECy7, rat-anti-mouse Siglec-F-PE, pan-leukocyte rat-anti mouse CD45-PerCP, and rat-anti-mouse CD11c-APC. Neutrophils were defined as CD45hi Ly6G+, eosinophils were defined as CD45hiLy6G− CD11c− SiglecF+, and alveolar macrophages were defined as CD45hi Ly6G− CD11c+SiglecF+. Flow cytometry was performed on a Guava EasyCyte 8HT (EMD Millipore).

### Total RNA processing and gene expression analysis

Lungs were removed and flash frozen in liquid nitrogen for RNA extraction. Total RNA was extracted from lungs homogenized in Trizol reagent (Invitrogen). A549 cells were lysed in Trizol, post stimulation, for RNA extraction. Following the aqueous upper phase separation further RNA purification was performed using Qiagen RNEasy column with on column DNAse treatment per manufacturer`s recommendations. For cDNA synthesis, five micrograms of total RNA were used using high-capacity cDNA synthesis kit (Life Technologies) according to manufacturer`s protocol. For qPCR, Power-Up SYBR Green PCR Master Mix (Applied Biosystems) was used with Mxp3500 Real-time PCR system (Agilent) or CFX Connect Real-Time PCR. Mouse B-Actin was used as housekeeping gene for cytokine analysis in murine model, whereas human GAPDH was used as housekeeping gene for cytokine analysis of human cell lines. Most of the PCR primers were designed using software from the whole sequence available at GenBank (National Center for Biotechnology Information). A list of murine primers used can be found in Table 1 and human primers in Table 2.

**Table 1:** Mouse primers.

#	Mouse primers		Sequence
**1**	Actin B	Left / Forward Primer:	5’ CTA AGG CCA ACC GTG AAA AG 3’ 5’ ACC AGA GGC ATA CAG GGA CA 3’
		Right / Reverse Primer:	
**2**	IL1a	Left / Forward Primer:	5’ TCT ATG ATG CAA GCT ATG GCT CA 3’ 5’ CGG CTC TCC TTG AAG GTG A 3’
		Right / Reverse Primer:	
**3**	IL1b	Left / Forward Primer:	5’ GCC CAT CCT CTG TGA CTC AT 3’ 5’ AGG CCA CAG GTA TTT TGT CG 3’
		Right / Reverse Primer:	
**4**	IL5	Left / Forward Primer:	5’ GAA GTG TGG CGA GGA GAG AC 3’ 5’ GCA CAG TTT TGT GGG GTT TT 3’
		Right / Reverse Primer:	
**5**	IL6	Left / Forward Primer:	5’ CTG CAA GAG ACT TCC ATC CAG 3’ 5’ AGT GGT ATA GAC AGG TCT GTT GG 3’
		Right / Reverse Primer:	
**6**	IL10	Left / Forward Primer:	5’ CTT ACT GAC TGG CAT GAG GAT CA 3’ 5’ GCA GCT CTA GGA GCA TGT GG 3’
		Right / Reverse Primer:	
**7**	IL12b	Left / Forward Primer:	5’ GTC CTC AGA AGC TAA CCA TCT CC 3’ 5’ CCA GAG CCT ATG ACT CCA TGT C 3’
		Right / Reverse Primer:	
**8**	IL13	Left / Forward Primer:	5’ CAG CTC CCT GGT TCT CTC AC 3’ 5’ CCA CAC TCC ATA CCA TGC TG 3’
		Right / Reverse Primer:	
**9**	IL22	Left / Forward Primer:	5’ CAA CTT CCA GCA GCC ATA CA 3’ 5’ GTT GAG CAC CTG CTT CAT CA 3’
		Right / Reverse Primer:	
10	IL23a	Left / Forward Primer:	5’ AAT AAT GTG CCC CGT ATC CAG T 3’ 5’ GCT CCC CTT TGA AGA TGT CAG 3’
		Right / Reverse Primer:	

**Table 2:** Human primers.

#	Human primers		Sequence
**1**	GAPDH	Left / Forward Primer:	5’ GAC CTG ACC TGC CGT CTA GAA AAA 3’ 5’ ACC ACC CTG TTG CTG TAG CCA AAT 3’
		Right / Reverse Primer:	
**2**	IL33	Left / Forward Primer:	5’ GTT GCA TGC CAA CAA CAA GGA 3’ 5’ GTT GCA TGC CAA CAA CAA GGA G 3’
		Right / Reverse Primer:	
**3**	Tslp	Left / Forward Primer:	5’ TAG CAA TCG GCC ACA TTG CC 3’ 5’ CTG AGT TTC CGA ATA GCC TG 3’
		Right / Reverse Primer:	
**4**	CCL11	Left / Forward Primer:	5’ ACA CCT TCA GCC TCC AAC AT 3’ 5’ GGT CTT GAA GAT CAC AGC TT 3’
		Right / Reverse Primer:	
**5**	IL1b	Left / Forward Primer:	5’ CCA CAG ACC TTC CAG GAG AAT G 3’ 5’ GTG CAG TTC AGT GAT CGT ACA GG 3’
		Right / Reverse Primer:	
**6**	IL6	Left / Forward Primer:	5’ CCA CAC AGA CAG CCA CTC ACC 3’ 5’ CTA CAT TTG CCG AAG AGC CCT C 3’
		Right / Reverse Primer:	
**7**	IL8	Left / Forward Primer:	5’ TGC AGC TCT GTG TGA AGG TGC AG 3’ 5’ TGT GTT GGC GCA GTG TGG TCC 3’
		Right / Reverse Primer:	
**8**	TNFa	Left / Forward Primer:	5’ CCC CCA GAG GGA AGA GTT CCC CA 3’ 5’ AGC GCT GAG TCG GTC ACC CT 3’
		Right / Reverse Primer:	
**9**	IL25	Left / Forward Primer:	5’ CCA GGT GGT TGC ATT CTT GG 3’ 5’ TGG CTG TAG GTG TGG GTT CC 3’
		Right / Reverse Primer:	
**10**	Ym1	Left / Forward Primer:	5’ ACT TTG ATG GCC TCA ACC TG 3’ 5’ AAT GAT TCC TGC TCC TGT GG 3’
		Right / Reverse Primer:	

### Data analysis methods

Analysis of mouse data was performed with FlowJo software (TreeStar). GraphPad Prism was used for generation of graphs and figures and for statistical analyzes (GraphPad Software). Unpaired t-tests were used to measure statistical significance when two groups were compared, and one or two-way analysis of variance (ANOVA) tests were used along with Tukey’s post-tests for multiple comparisons, respectively. Differences between experimental groups that resulted in a p-value of less than 0.05 were considered significant. Analysis of mouse flow cytometric data was performed with FlowJo software, version 10 (Becton-Dickinson).

## Results

### 1. *In vitro* cytokine modulation due to FIBCD1 in response to chitin of varying sizes

**Fig 1:**
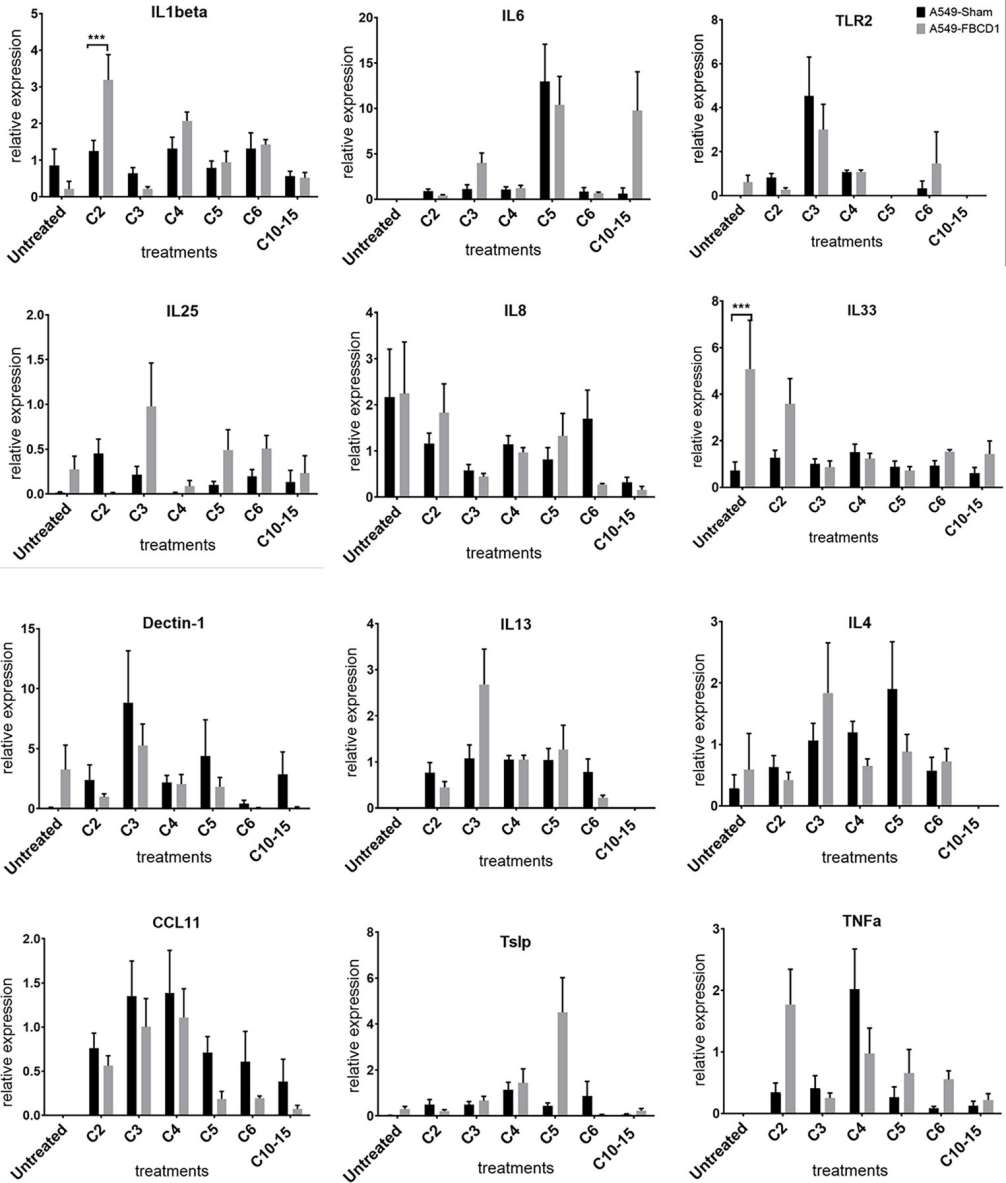
Cytokine modulation in human lung epithelial cells over-expressing FIBCD1 in response to chitin fragments of different sizes. A549-Sham transfected cells are used as control. Cells stimulated for 6 hours and harvested as described in materials and methods and analyzed by qRT-PCR for expression of indicated cytokines using ΔΔ Ct method. Data are a summary of two-three experiments. n = 12/group. *p<0.05, **p<0.01, ***p<0.001, ****p<0.0001.

It has been demonstrated previously that stimulation with different chitin particle sizes results in size-dependent immune responses [13, 17]. Hence, the role of FIBCD1 in response to different chitin particle sizes is a matter of interest. To examine this role, we used qRT-PCR of chitin-stimulated A549 lung epithelial cells overexpressing FIBCD1 (sham transfected cells as control) to determine FIBCD1-mediated modulation of cytokine expression in response to chitin. A pattern of decreased inflammatory cytokines IL-1β and TNF were observed in FIBCD1-expressing cells, after stimulation with increasing oligomer length of chitin, yet IL-1 β expression was significantly higher in FIBCD1-expressing cells after C2 dimer stimulation when compared to non-expressing lung epithelial cells (Fig 1). A pattern of decreased epithelial cytokine IL-33 was also observed when FIBCD1 overexpressed cells were stimulated with chitin fragments of increasing sizes. Moreover, for certain cytokines mRNA induction appeared to decrease with increasing oligomer length. For example, for IL-1β and TNF, chitobiose (C2) induced higher mRNA levels than longer oligomers (e.g. C10-15), except for chitotriose (C3) in TNF. However, for other cytokines such as IL-6 and TSLP, longer oligomers were more successful in stimulating expression. These results show that differences between FIBCD1 overexpressing and sham cells due to chitin stimulation were discernible, suggesting that over expression of FIBCD1 modulates inflammatory and epithelial cytokine expression in lung epithelial cells when stimulated by different sizes of chitin fragments.

### 2. *In vitro* cytokine modulation due to FIBCD1 in response to different strains of *Aspergillus fumigatus*

**Fig 2:**
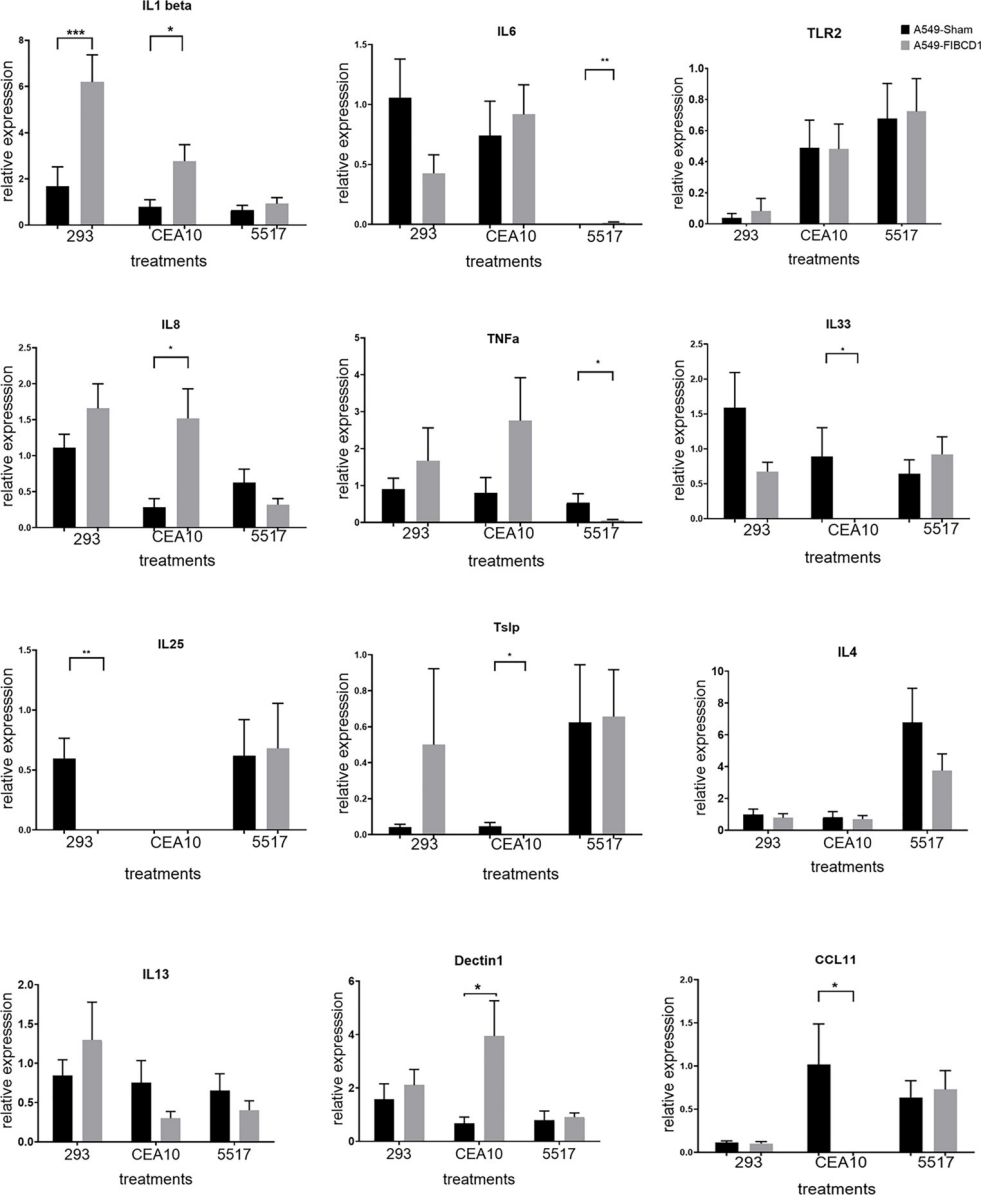
Cytokine modulation in human lung epithelial cells over-expressing FIBCD1 in response to different strains of *Aspergillus fumigatus*. A549-Sham transfected cells are used as control. Cells stimulated and harvested as described in materials and methods and analyzed by qRT-PCR for expression of indicated cytokines using ΔΔ Ct method. Data are a summary of two-three experiments. n = 12/group. *p<0.05, **p<0.01, ***p<0.001, ****p<0.0001.

Different fungal strains have different virulence levels and antigenic composition. To further explore the role of FIBCD1, we examined how FIBCD1 modulated the response to different fungal strains (namely, Af293, CEA10 and 5517), in lung epithelium. IL-1β was increased in FIBCD1-expressing lung epithelial cells significantly in response to fungal conidia of different strains of *Aspergillus fumigatus* (Fig 2). But expression levels of other inflammatory or epithelial cytokines varied between strains. Stimulation of cytokine responses was strain-variable in the presence or absence of FIBCD1. Af5517 was generally less affected by the presence of FIBCD1, whereas Af293 and CEA10 showed generally striking differences between FIBCD1 overexpressed or sham cells.

### 3. *In vivo* cytokine modulation due to FIBCD1 in response to chitin of varying sizes

**Fig 3:**
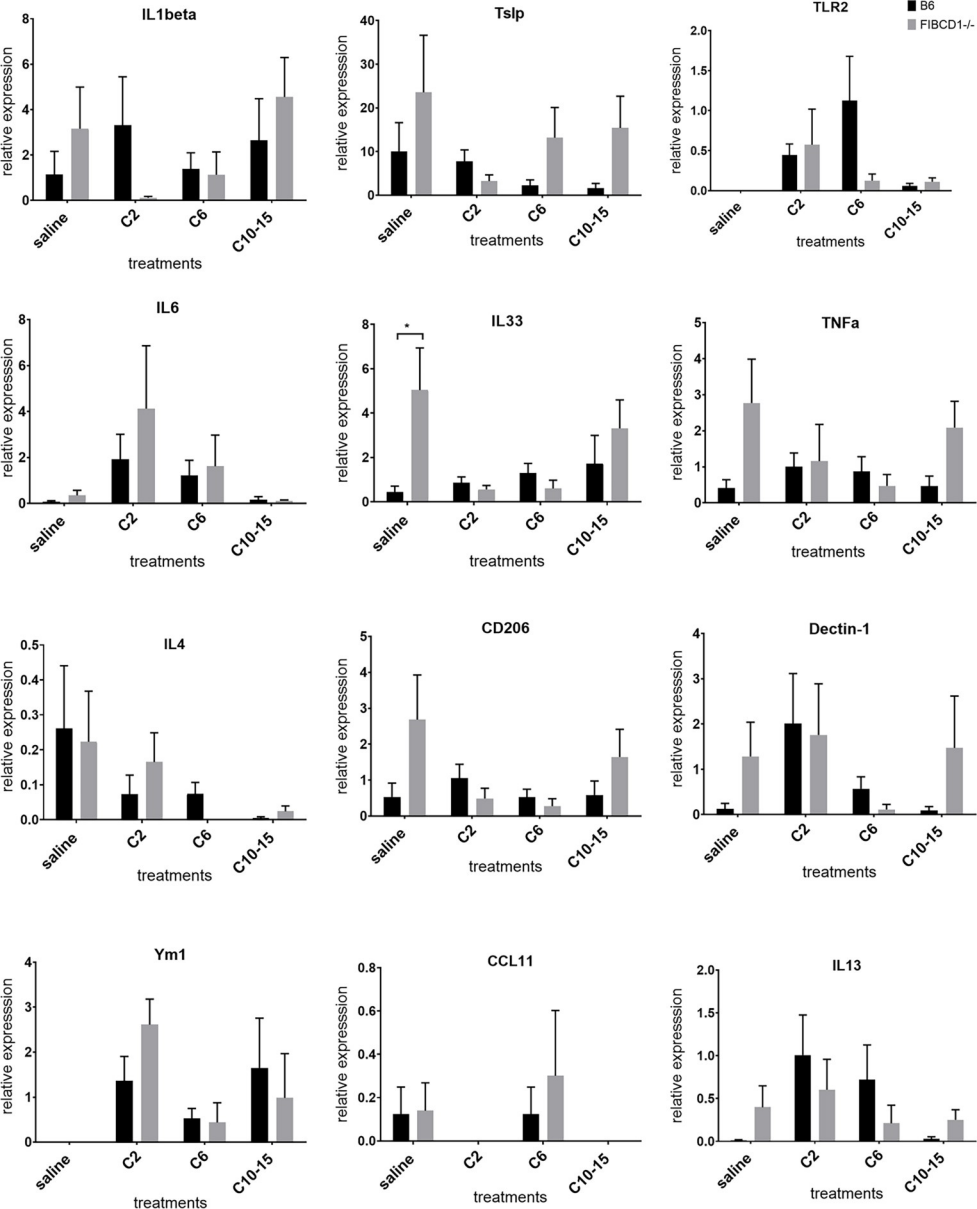
Cytokine modulation *in vivo* due to FIBCD1 in response to chitin fragments of different sizes. Mice were challenged with chitin fragments (as described in materials and methods) and analyzed by qRT-PCR for expression of indicated cytokines using ΔΔ Ct method. Data are a summary of two experiments. n = 8/group. *p<0.05.

To explore the role of FIBCD1 further *in vivo*, lungs of chitin-challenged mice of wild-type and Fibcd1-/—mice were analyzed to determine FIBCD1-mediated modulation of cytokine expression in response to chitin. Loss of FIBCD1 *in vivo* lead to an increase in inflammatory cytokine mRNA like IL1β and TNF upon stimulation with increasing size of chitin (Fig 3). A decreased expression of epithelial cytokines IL33, was observed when stimulated with chitin dimer, in the absence of constitutive expression of FIBCD1. Although no clear pattern of cytokine modulation was evident in the lungs of wild-type and Fibcd1-/- mice, we did notice that chitin dimers and oligomers seemed to have opposite effects on certain inflammatory cytokines like IL1β, IL6 and on certain epithelial cytokines like Tslp and IL33 in the lungs of FIBCD1 knockout mice (Fig 3). A gradual decrease in expression of IL4, lL6 and IL13 was observed in the absence of FIBCD1, with increasing size of chitin fragments. Expression of TLR2, a key candidate known to modulate downstream signalling pathways in response to chitin oligomers, was observed to decrease with increasing size of chitin, in absence of FIBCD1. Thus, FIBCD1 differentially modulates inflammatory and epithelial cytokine expression when stimulated by different sizes of chitin fragments, in a model -specific manner.

### 4. Effect of FIBCD1 on PMN cell recruitment in response to chitin of varying sizes in a murine model

**Fig 4:**
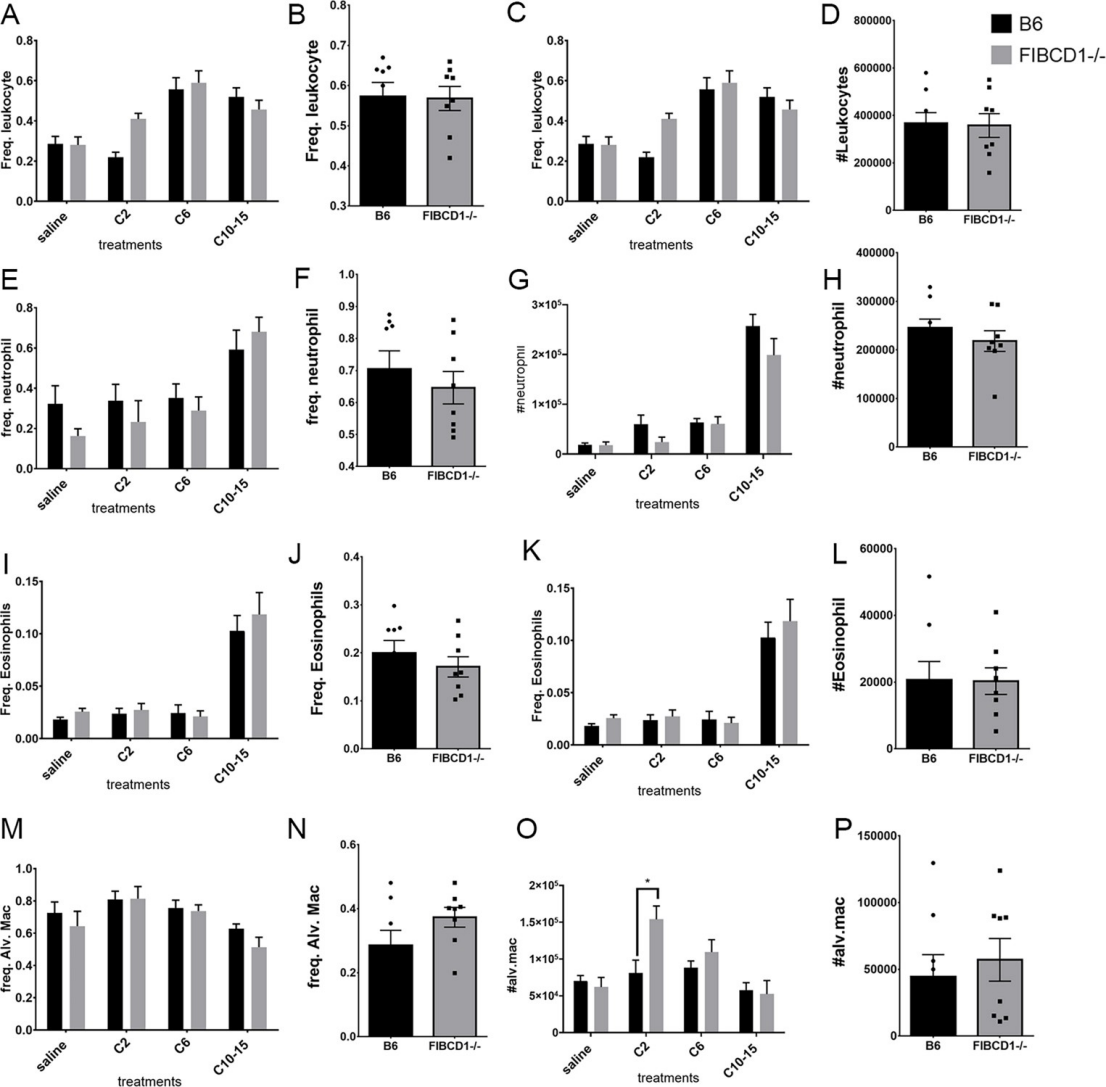
Airway Immune cells recruitment in BALF in response to aspiration of different sizes of purified chitin. Graphical representation depicting gating of BALF CD45+ leukocytes of purified chitin (A, C) and of crude chitin particles (B, D); CD45+ Ly6G- neutrophils of purified chitin (E,G) and of crude chitin particles (F,H); CD45+ Ly6G-SiglecF+CD11c- eosinophils of purified chitin (I, K) and of crude chitin particles (J,L); CD45+ Ly6G-SiglecF+CD11c+ alveolar macrophages of purified chitin (M, O) and of crude chitin particles (N,P). Data are a summary of 2 experiments. n = 8/group. *p<0.05, **p<0.01, ***p<0.001, ****p<0.0001.

Chitin is known to modulate inflammatory cells in BALF in an allergy model [13, 19]. Yet the effect of FIBCD1 on airway inflammation triggered by different sizes of chitin has not been explored. To determine how the size of chitin influences PMN cell recruitment in absence of FIBCD1 in chitin-challenged mice, mice of both groups were challenged with different sizes of purified chitin (dimer, hexamer and oligomer) and crude chitin particles. BALF were collected from mice of both groups 6 hours post-challenge and stained for PMN cells and analyzed by flow cytometry. Consistently with [12], an overall increase in leukocytes is observed, especially in neutrophils and eosinophils, in both wild type and knockout mice in response to increasing size of chitin fragments (Fig 4C, 4G and 4K). Presence or absence of FIBCD1 did not affect this overall increase in leukocytes. A decrease is noted in the level of alveolar macrophage population in the absence of FIBCD1, with increasing sizes of chitin oligomers (Fig 4O). Thus, chitin inhalation, particularly with C10-15 oligomers, modulates airway leukocyte recruitment independent of FIBCD1 expression.

### 5. *In vivo* cytokine modulation due to FIBCD1 in response to different strains of *Aspergillus fumigatus*

**Fig 5:**
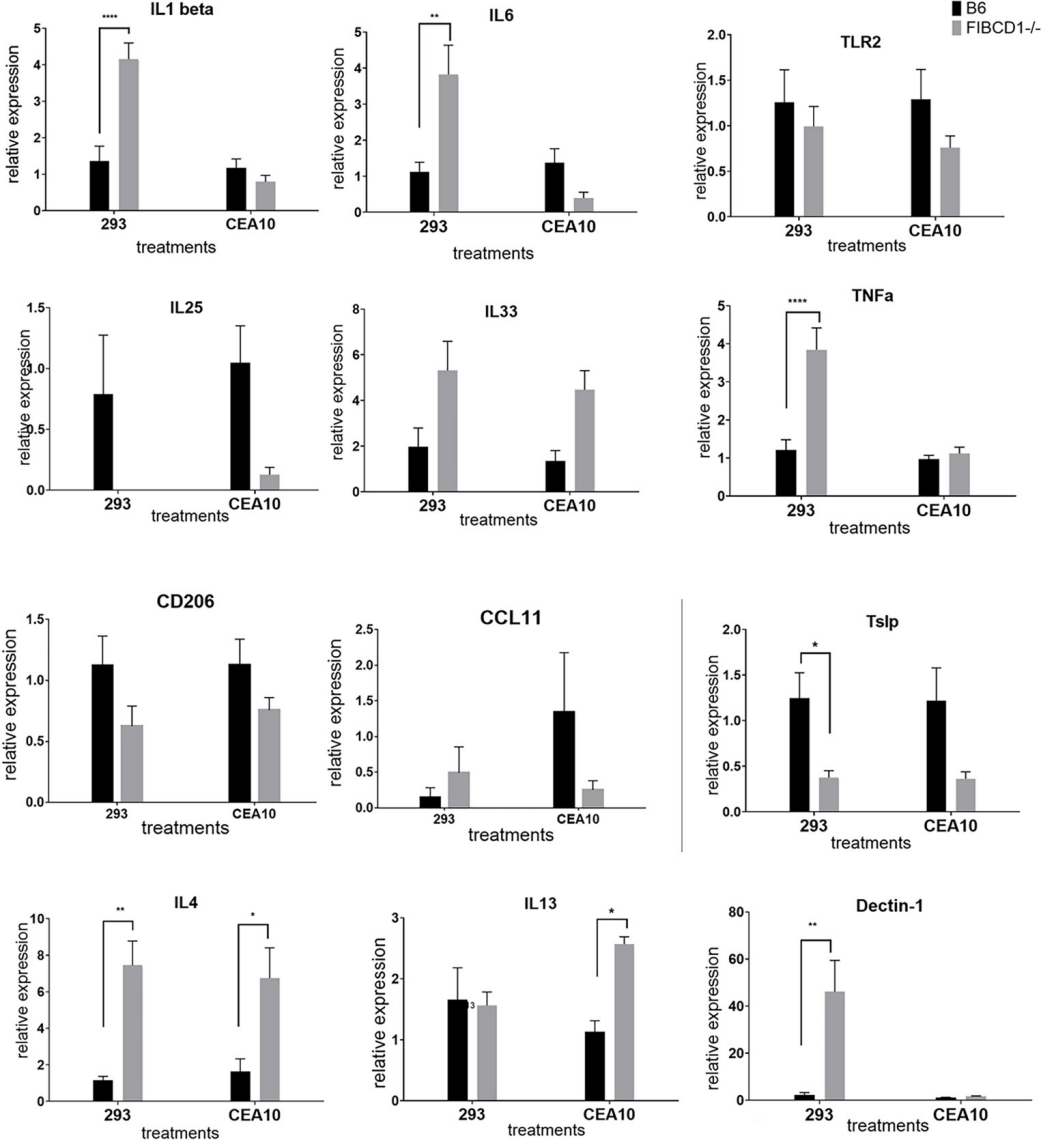
Cytokine modulation *in vivo* due to FIBCD1 in response to different strains of *Aspergillus fumigatus*. Mice were challenged with high dose of fungal conidia (as described in materials and methods).and samples are analyzed by qRT-PCR for expression of indicated cytokines using ΔΔ Ct method. Data are a summary of two experiments. n = 8/group. *p<0.05, **p<0.01, ***p<0.001, ****p<0.0001.

Different fungal strains have different virulence levels and antigenic composition. To complement the *in vivo* analysis of purified chitin oligomers, we also examined how FIBCD1 modulated the response to the different aforementioned *A*. *fumigatus* strains 293 and CEA10 *in vivo*. Absence of FIBCD1 led to an increase in IL1β, IL6 and TNF mRNAs in murine lung with fungal strain Af293 (Fig 5). A decrease in the transcription of the epithelial cytokines IL25, and Tslp was observed, with absence of FIBCD1 in response to different fungal strains. Similar to results from our *in vitro* model (Fig 2), we observed modulation of IL6 mRNA levels by FIBCD1 expression. Consistent in both the models, we observe similar trend of increased IL6 mRNA expression in absence of FIBCD1 compared to decreased IL6 with FIBCD1 expression *in vitro* (Fig 2). Thus, modulation of inflammatory cytokines by FIBCD1 in response to virulence level in fungal strains was observed, with results that were specific to *in vitro* and *in vivo* models.

### 6. Role of FIBCD1 on immune cell recruitment in response to different strains of *Aspergillus fumigatus*

**Fig 6:**
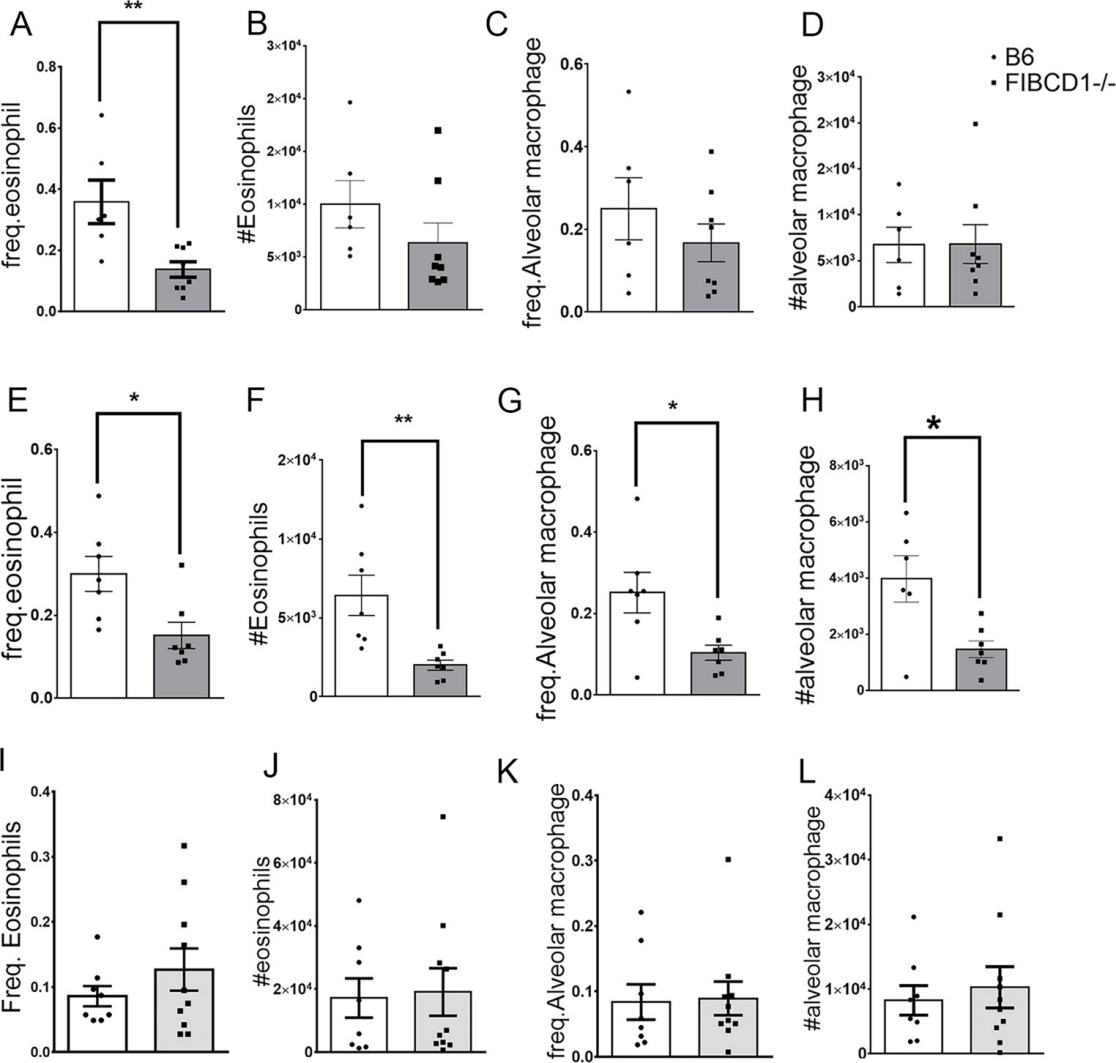
Differences in recruitment of inflammatory cells, in absence of gene, when challenged with different strains and dosage of *Aspergillus* conidia. Mice of both groups were challenged with either single high dose- 50x10^6 conidia- of two different strains of *Aspergillus* sp.-Af293 (A-D) and AfCEA-10 (E-H), or single low dose - 5x10^6- of high chitin-rich strain- Af5517 (I-L). BALF were analyzed for PMN cells recruitment. Representative flow plots depicting gating of CD45+Ly6G-SiglecF+CD11c-BALF eosinophils (A-B, C-D), CD45+Ly6G-SiglecF+CD11c+ Alveolar macrophage (C-D, E-F). Data shown are a summary of two-three experiments. *p<0.05, **p<0.01.

To assess the immediate effect of FIBCD1 on recruitment of immune cells in response to chitin, wild type and knockout mice were challenged with either single dose- 50x10^6 conidia- of two different strains of *Aspergillus* sp.-Af293 and CEA-10, or single dose - 5x10^6- of high chitin-rich strain- Af5517 and harvested 24 hours post challenge for BALF and lungs. BALF cells were stained for PMN and analyzed by flow cytometry. Although total number of eosinophils do not change significantly (Fig 6B), frequency of eosinophil levels significantly drops in absence of FIBCD1 in response to challenges by both Af293 (Fig 6A), and CEA10 (Fig 6E and 6F). Alveolar macrophages as well show a significant decrease in the absence of FIBCD1 in response to challenges by CEA10 (Fig 6G and 6H), but not when challenged by Af293 (Fig 6C and 6D). Single low dose of high chitin-rich strain- Af5517 does not exert any significant changes between the experimental groups (Fig 6I–6L). Thus, fungal challenge of Fibcd1-/- mice resulted in decreased recruitment of eosinophils or macrophages, regardless of strain.

## Discussion

In response to chitin fragments, overexpression of FIBCD1 leads to a decrease in the inflammatory cytokines IL1β, TNF with increasing size of chitin oligomers, although these cytokines were increased relative to the levels that were observed in control, sham-transfected cells. An absence of FIBCD1 in mice leads to an overall increase in inflammatory cytokines in response to aspiration of increasing size of chitin fragments, with fungal strain-specific modulation of inflammatory cytokines. Although we did not measure protein expression levels of the cytokines and receptors, previous works on A549 cells have found correlation between mRNA and their corresponding protein expression levels [23, 24]. The differences that we observed in *in vivo* and *in vitro* results are supported by prior studies [5, 25]. Chitin stimulated M2 macrophage activation *in vivo*, but not *in vitro*, resulted in increased TNF expression [5].

Epithelial cytokines are important immune modulators that are stimulated by chitin. They are major candidates to activate ILC2s in response to chitin, indirectly activating Th2 cytokines and eosinophils, mucus secretion, and inflammation [7]. In this study, when FIBCD1 is expressed *in vitro* in A549 epithelial cells, we observed an overall decrease in specific epithelial cytokines in response to chitin fragments of increasing size or different fungal strains with varied chitin content or levels of virulence. Here we observe two different phenotypes in different models of chitin exposure. The source of chitin administered is an important aspect to consider, as immune modulation by FIBCD1 varies when exposed to pure chitin fragments compared to exposure to the chitin containing cell wall of different fungal strains.

This is not altogether surprising as the fungal cell wall is composed of layers of glucan, mannan, and chitin, all of which can stimulate different PRRs on either epithelial or immune cells. Hence immune responses to chitin are combined with recognition and responses to glucan and mannan when fungal cells are used for stimulation [3, 4]. Furthermore, full fungal pathogens may employ immune evasion strategies [26–28]. When chitin-containing pathogens enter a host, the innate immune responses release oxidants and chitinases leading to chitin fragmentation. The intermediate-sized fragments induce inflammation through NF-KB signalling. More fragmentation continues and smaller particles of chitin are generated that induce anti-inflammatory effects through IL10 production to control local inflammation [5]. Hence, different sizes of chitin initiate different immune responses. The difference in inflammatory responses stimulated by different sized fragments is due to combined recognition of TLR2, dectin-1, and mannose receptors [12]. The source of chitin administered is an important aspect to consider, as immune modulation by FIBCD1 varies when exposed to pure chitin fragments compared to exposure to different fungal strains. This might explain the observed differences in cytokine expression profiles. We also observed differences in inflammatory cell recruitment in BALF *in vivo*. When mice were challenged with either purified chitin fragments of increasing size or with different fungal strains with varying virulence and/or cell wall composition, absence of FIBCD1 resulted in an increase in leukocyte populations with increasing size of chitin oligomers, particularly in neutrophil and eosinophil populations. The observed differences in this study suggest two different immune pathways stimulated by chitin. Reese et al. observed chitin-induced eosinophil recruitment, aided by chitin-induced AAM, initiating Th2 cytokine production and type 2 inflammatory responses, when pure chitin particles were administered directly into the lungs of mice [8]. But interestingly, chitin has been known to stimulate type 1 cytokines as well [29]. Shibata et al. showed that chitin, when administered orally, inhibited allergen- induced IgE production and lung inflammation and resulted in a decrease in type 2 cytokines (in *in vitro* cultures) and these inhibitory effects were mediated by IFN-y produced by NK cells. In other studies, it was shown that chitin is a strong Th1 adjuvant that can downregulate Th2 immunity [15, 17, 29].Similar results have been observed to the allergic response by *A*. *fumigatus*, when chitin micro particles were administered orally [17].

TSLP and Arg1 are considered as components of Th2 polarization and are blocked at the site of allergen-induced inflammation by water soluble chitosan, the deacetylated form of chitin [8]. We observed similar characteristics of both Th1-skewed and Th2- specific responses, in response to chitin fragments or in response to fungal strains, in BALF of challenged mice in the *in vivo* model. Analyzing the lavage fluid cells of mice challenged with chitin fragments with increasing size, we observed an overall increase in leukocyte population in absence of FIBCD1, especially an increase in eosinophil and neutrophil populations. The inflammatory cytokines showed an increase in absence of FIBCD1 as well. It is possible that this increase resulted in a spike in eosinophils and other innate cells which is due to chitin-induced alternative macrophage activation, and that could lead to activation of ILC2 and Th2 cytokines thereby triggering Type 2 immunity [7]. Interestingly, an opposite phenotype is observed when mice were challenged with different fungal strains of varied virulence or cell wall composition. Analyzing the BALF of challenged mice, an absence of FIBCD1 resulted in a decrease in inflammatory cells in BALF, with a notable decrease in eosinophils and alveolar macrophages. A decrease in lung inflammatory cytokines was also observed in the absence of FIBCD1. It is likely to be more of a Th1-skewed anti-inflammation that occurs in absence of FIBCD1 in this model. This phenomenon has been observed in earlier studies as well, where chitin administration to allergic mice takes a Th1-skewed immune pathway via IFNy [17]. Also, it has been speculated that FIBCD1 might modulate a distinct yet not fully understood immune response against *A*. *fumigatus*, especially via opsonization and chemo attraction of neutrophils to the site of infection [30–32].

## Conclusions

FIBCD1 expression modulated immune responses to chitin, in both *in vitro* and *in vivo* models. But the exact mechanism of action is yet to be defined. It will be important to define downstream pathways altered by FIBCD1 in future studies. Interestingly, chitin has been known to enhance survival in *Candida albicans* infection, if the mice were pre-treated with chitin prior infection [33].Chitin has also been known to enhance T cell function, NK cell activity and oxidative burst of macrophage which help in defense and dissolution of threat [8, 34, 35]. This host-favouring role of chitin is an interesting aspect to probe further, since FIBCD1 has been found to be host-detrimental in lung fungal infection [22], and FIBCD1 also regulates fungal colonization in gut and reduce disease severity in case of acute injury model in gut [19].
